# Aquaculture Can Promote the Presence and Spread of Antibiotic-Resistant Enterococci in Marine Sediments

**DOI:** 10.1371/journal.pone.0062838

**Published:** 2013-04-26

**Authors:** Andrea Di Cesare, Gian Marco Luna, Carla Vignaroli, Sonia Pasquaroli, Sara Tota, Paolo Paroncini, Francesca Biavasco

**Affiliations:** 1 Department of Life and Environmental Sciences, Polytechnic University of Marche, Ancona, Italy; 2 Institute of Marine Sciences, National Research Council, Venezia, Italy; Missouri University of Science and Technology, United States of America

## Abstract

Aquaculture is an expanding activity worldwide. However its rapid growth can affect the aquatic environment through release of large amounts of chemicals, including antibiotics. Moreover, the presence of organic matter and bacteria of different origin can favor gene transfer and recombination. Whereas the consequences of such activities on environmental microbiota are well explored, little is known of their effects on allochthonous and potentially pathogenic bacteria, such as enterococci. Sediments from three sampling stations (two inside and one outside) collected in a fish farm in the Adriatic Sea were examined for enterococcal abundance and antibiotic resistance traits using the membrane filter technique and an improved quantitative PCR. Strains were tested for susceptibility to tetracycline, erythromycin, ampicillin and gentamicin; samples were directly screened for selected tetracycline [*tet*(M), *tet*(L), *tet*(O)] and macrolide [*erm*(A), *erm*(B) and *mef*] resistance genes by newly-developed multiplex PCRs. The abundance of benthic enterococci was higher inside than outside the farm. All isolates were susceptible to the four antimicrobials tested, although direct PCR evidenced *tet*(M) and *tet*(L) in sediment samples from all stations. Direct multiplex PCR of sediment samples cultured in rich broth supplemented with antibiotic (tetracycline, erythromycin, ampicillin or gentamicin) highlighted changes in resistance gene profiles, with amplification of previously undetected *tet*(O), *erm*(B) and *mef* genes and an increase in benthic enterococcal abundance after incubation in the presence of ampicillin and gentamicin. Despite being limited to a single farm, these data indicate that aquaculture may influence the abundance and spread of benthic enterococci and that farm sediments can be reservoirs of dormant antibiotic-resistant bacteria, including enterococci, which can rapidly revive in presence of new inputs of organic matter. This reservoir may constitute an underestimated health risk and deserves further investigation.

## Introduction

The spread of antibiotic-resistant microorganisms in the environment is widely recognized as an important public health issue, and there is concern on the future ability to treat infectious diseases. Contaminated seawater and sediments can become reservoirs of virulent and antibiotic-resistant strains of fecal bacteria [1], [2], [3], including enterococci [4], which are capable of transmitting resistance genes to other bacteria by horizontal gene transfer mechanisms, thus contributing to dissemination of resistance genes into the marine environment.

The presence of resistant bacteria raises particular concern at fish-farm sites, where a large use of antibiotics has been made in recent years [2]. Resistant bacteria can reach aquaculture sites also *via* agricultural and urban wastewaters; these contain the typical intestinal flora and pathogens of animals and humans, which are usually resistant to antibiotics [5]. These emerging contaminants can accumulate in the underlying sediments, where they interact with the benthic microbial communities [6]. Even in absence of continuous antimicrobial administration, resistant microorganisms can persist in protected reservoirs such as sediments or fish gut [4], [7]. Sediments are a particularly favorable environment for benthic allochthonous bacteria since they provide nutrients and protection from biotic and abiotic stress, allowing their long-term persistence in a culturable state or even their re-growth [8], [9], [10].

Enterococci are part of the human and animal intestinal microflora and are used as fecal indicator bacteria (FIB) for monitoring recreational waters and for assessing potential risks for human health [11], [12]. They have been recognized as major agents of nosocomial infections [13], [14] whose treatment is often complicated by antibiotic resistance (AR), either intrinsic and acquired [15]. Acquired AR is mainly due to integration of external genetic material mediated by transposon or plasmid transfer [16], [17].

A greater understanding of the stress-resistance ability of *Enterococcus* species, virulence traits and AR is required for a full appreciation of the complexity of *Enterococcus* species in causing human disease [18]. While a number of papers have documented the presence, fate and reservoirs of enterococci in coastal marine systems and other aquatic environments [11], [19], [20], [21], [22], little information is available on the distribution of resistant enterococci and their determinants at aquaculture sites [4], [23].

In this study, sediment samples were analyzed to investigate the impact of fish aquaculture on the spread and abundance of tetracycline-, macrolide-, β-lactam- and aminoglycoside-resistant benthic enterococci. Both culture-dependent and molecular tools were used to quantify enterococcal abundance and directly search for resistance genes. *In vitro* enrichment assays in the presence of antibiotics were also carried out to investigate the possible consequences of their release into the marine environment, with emphasis on the abundance of benthic enterococci and the profile of resistance genes, which are potentially transmissible between both autochthonous and allochthonous bacterial species.

## Materials and Methods

### Ethics Statement

All necessary permits were obtained for the described field studies. The approval for sediment sampling was obtained from the owner of the private aquaculture facility, who wishes to remain anonymous. The sampling activities were not performed in a protected area and they did not involve invertebrates, plant species, corals or fish.

### Site, Sediment Sampling and Environmental Variables

**Figure 1:**
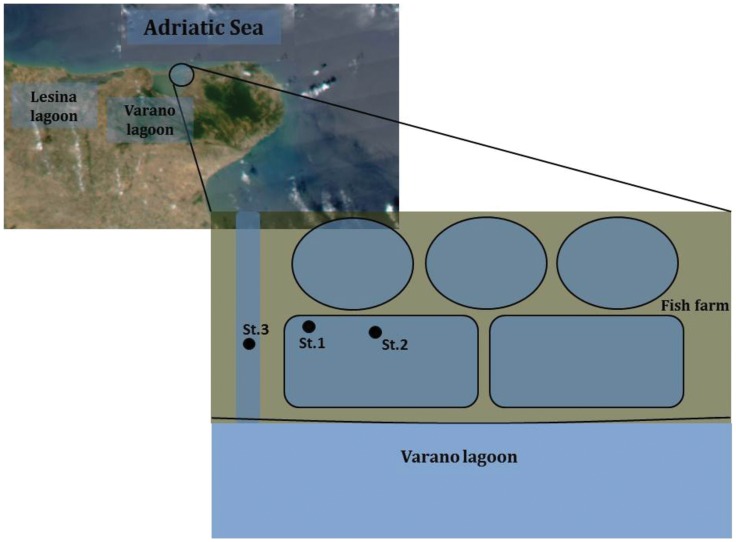
Location of the fish farm and of the sampling stations. The map is from http://earthobservatory.nasa.gov/, image courtesy Jesse Allen.

Sediments were collected in June 2011 at a fish farm in Varano lagoon (central Italy; Figure 1). The farm consists of several ponds receiving water from the lagoon through a canal. Samples were collected from 3 stations in the largest pond (latitude 41° 54′ 33.37″ N; longitude 15° 45′ 8.91″ E), which measured 54×21×2 m and hosted about 14,000 seabream and seabass (data provided by the owner). Station (St.) 1 was located in an area of the pond used for feed administration; St. 2 was still in the pond but far from the feeding area; and St. 3 was upstream, in the water supply canal connecting the pond to the lagoon, and was thus unaffected by farming activities (control station). The water temperature was 29°C. The farm owner denied all antibiotic use in the pond, either for therapeutic or for growth promotion purposes, and reported using exclusively non-medicated feed (Hendrix, Verona, Italy). Sediments were collected using sterile Plexiglas corers, placed in sterile containers, and stored in the dark until delivery to the laboratory (max 5 h). Sub-samples were used for cultural and molecular microbiological analyses and to determine grain size, (by the sieving technique) and total organic matter, which was determined as the difference between dry weight (60°C, 48 h) of the sediment and the weight of the residue after combustion for 2 h at 450°C [8].

### *Enterococcus* spp. Isolation and Enumeration

The membrane filter (MF) technique was used for the enumeration of culturable *Enterococcus* spp. Briefly, 30 g of sediment from each station was suspended in 300 ml of saline solution, vigorously shaken and sonicated to detach bacteria as described previously [1]. The supernatant was pre-filtered through a 30 µm membrane; 10 ml aliquots of the suspension and 1/10 dilutions were filtered (0.2 µm pore size), and filters were placed on Slanetz-Bartley plates (SB; Oxoid, Basingstoke, UK) and incubated for 48 h at 37°C. Grown colonies were counted and the abundance of *Enterococcus* spp. was expressed as CFU/g of wet sediment. Selected colonies were further amplified on SB plates and incubated for 48 h at 42°C. To establish if they belonged to the genus *Enterococcus* amplified cultures were tested for growth at 42°C in the presence of 6.5% NaCl.

### Antibiotic Susceptibility Testing

Minimum Inhibitory Concentrations (MIC) were determined by broth microdilution according to CLSI guidelines [24]; the results were interpreted according to CLSI M100-S21 (2011). *Enterococcus faecalis* ATCC 29212 was used as the control strain. Tetracycline (TET), erythromycin (ERY), ampicillin (AMP) and gentamycin (CN) were purchased from Sigma-Aldrich (Saint Louis, MO, USA).

### *In vitro* Enrichment by Sediment Incubation in Rich Broth Supplemented with Antibiotics

Aliquots of sediment from the 3 stations were incubated in rich medium in the presence of one of the four antibiotics. Four sterile bottles per station were prepared, each containing 5 g of sediment and 50 ml Brain Hearth Infusion (BHI) broth (Oxoid) added with TET (10 µg/ml), ERY (20 µg/ml); AMP (20 µg/ml) or CN (250 µg/ml). Antibiotic concentrations were those generally used to select antibiotic-resistant isolates [4]. After 24 h incubation at 37°C DNA was extracted from sediment and broth.

### DNA Extraction and Purification

DNA was extracted using different protocols depending on sample type. The commercial kits Ultra Clean Mega Soil DNA Isolation (MoBio, Carlsbad, CA, USA) and Fast DNA SPIN Kit for Soil (Q•BIO Gene, Fountain Parkway Solon, OH, USA) without (10 g aliquots) and with antibiotic enrichment (0.5 g aliquots) were used for farm sediments, whereas the procedure described by Hynes et al. (1992) was used for the antibiotic-enriched broth cultures [25]. DNA extracts to be used undiluted were further purified with the Wizard SV Gel and PCR Clean-Up System (Promega, Madison, WI, USA) to remove PCR inhibitors.

### PCR Detection of Resistance Genes

Two Multiplex-PCR assays were developed to detect simultaneously *tet*(M), *tet*(L) and *tet*(O) and *erm*(B), *erm*(A) and *mef*, respectively. Three new primer pairs were designed to detect *tet*(O), *erm*(A) and *erm*(B) genes. For each target gene, several sequences deposited in the NCBI database were converted into FASTA format using the *NCBI Genome Workbench* software (http://www.ncbi.nlm.nih.gov/tools/gbench/), aligned using the *ClustalXII* software (http://www.clustal.org/), and the primers were designed on the conserved regions using the *NetPrimer* software (http://www.premierbiosoft.com/netprimer/index.html). Those showing the highest specificity by BLAST analysis were selected. PCR assays were performed in a final volume of 50 µl containing 5 µl of DNA (diluted 100 times or undiluted and purified) using a T Personal thermal cycler (Biometra, Göttingen, Germany). The PCR cycling program was as follows: 95°C for 10 min, followed by 35 cycles at 94°C for 30 s, 53°C [*tet*(M), *tet*(L), *tet*(O)] or 54°C [*erm*(B), *erm*(A), *mef*] for 30 s, 72°C for 90 s and final extension at 72°C for 7 min. Each mix contained 600 µM dNTPs, 6 mM MgCl_2_, 1× Buffer, 0.5 µM of each primer [1 µM of those targeting *tet*(M)], and 1.25 U hot-start Taq DNA polymerase (AmpliTaq Gold, Applied Biosystem, Foster City, CA, USA). The resistance genes *bla*Z and *aac (6′)-Ie aph (2*″*)-Ia* were sought as previously described [26]. The control strains and primer pairs used in PCR assays are reported in Tables 1 and 2, respectively.

**Table 1:** Control strains used in PCR assays.

Bacterial strain	Resistance gene(s)	Reference or source
*E. faecium* CM 4·2E	*tet*(M),*tet*(L),*erm*(B)	[4], [44]
*E. faecalis* PM 2·2T	*tet*(M), *tet*(L)	[4], [44]
*E. faecium* CF 2·1E	*tet*(M),*tet*(L),*erm*(B)	[4], [44]
*S. aureus* MU 50	*erm*(A)	ATCC^a^
*S. aureus*29213	*blaZ*	ATCC^a^
*S. pyogenes* 7008	*tet*(O),*mef*	[45]
*E. faecium M48*	*aac (6′)-Ieaph (2″)-Ia*	[26]

a American Type Culture Collection.

**Table 2:** Primer pairs used to detect resistance genes in PCR assays.

Target gene	Primer sequence (5′→3′)	Product size (bp)	Reference
*tet*(M)	1-GTTAAATAGTGTTCTTGGAG 2-CTAAGATATGGCTCTAACAA	657	[46]
*tet*(L)	1-CATTTGGTCTTATTGGATCG 2-ATTACACTTCCGATTTCGG	475	[46]
*tet*(O)	1-AGGGGGTTCTTTATGGCTG 2-CGTGAGAGATATTCCTGCG	223	This study
*erm*(B)	1-CCGAACACTAGGGTTGCTC 2-ATCTGGAACATCTGTGGTATG	139	This study
*erm*(A)	1-TAACATCAGTACGGATATTG 2-AGTCTACACTTGGCTTAGG	200	This study
*mef*	1- AGTATCATTAATCACTAGTGC 2-TTCTTCTGGTACTAAAAGTGG	348	[47]
*blaZ*	1-ACTTCAACACCTGCTGCTTTC 2-TAGGTTCAGATTGGCCCTTAG	240	[26]
*aac (6′)-Ie aph (2*″*)-Ia*	1-GAGCAATAAGGGCATACCAAAAATC 2-CCGTGCATTTGTCTTAAAAAACTGG	505	[48]

### Enumeration of Enterococci by Real Time Quantitative PCR (qPCR)

A Real Time Quantitative PCR (qPCR) assay was used to determine *Enterococcus* spp. abundance. The standard calibration curve was generated using a purified 23S rDNA amplicon obtained by a PCR reaction performed using DNA from *E. faecalis* ATCC 29212 and primers ECST748F and ENC854R, as previously described [8]. The 23S amplicon was purified by Gene Elute PCR Clean-up (Sigma-Aldrich) and quantified using an ND-1000 Nanodrop (Thermo Scientific, Wilmington, NC, USA). qPCRs were performed using the iCycler iQ-5 (Biorad, Hercules, OR, USA) in a 25 µl volume containing 2.5 µl of sample DNA, 0.2 µM of each (ECST748F and ENC854R), 12.5 µl of iQ™ SYBR® Green Supermix (Biorad), and Milli Q water (Millipore, Billerica, MA, USA) to reach the final volume. The amplification reaction was as follows: 95°C for 3 min, followed by 35 cycles at 95°C for 15 s, 60°C for 30 s and 72°C for 15 s. Melt curve analysis was carried out from 59°C to 95°C, with increments of 0.5°C/10 s. Suitable dilutions (i.e. containing from 10^−6^ to 10^−9^ ng of DNA) of 23S rDNA of the purified amplicon of *E. faecalis* ATCC 29212 were used for construction of the standard curve. Similar PCR reactions using DNA from sediment samples, either diluted and undiluted to account for potential qPCR inhibition [8], [27], were run together. These analyses consistently showed that undiluted DNA extracts were inhibited, as demonstrated by a threshold cycle (Ct) delay between qPCR results on this DNA extract and serial 10-fold dilutions (1∶10 and 1∶100). While the expected Ct difference between 10-fold dilutions in the absence of inhibition is 3.32, in our samples it was typically between 1 and 2 cycles less than expected without inhibition (data not shown). Each reaction was performed in triplicate. Reproducibility of the qPCR reaction was assessed by determining intra- and interassay repeatability of the standard curve. The coefficient of variation (CV) to evaluate intra-assay repeatability was calculated on the basis of the Ct value, by testing in triplicate the 4 dilutions containing from 10^−6^ to 10^−9 ^ng DNA of the target gene. The CV for interassay reproducibility was calculated based on the Ct value of the 4 dilutions in four different analysis sessions. The Limit of Detection (LOD) was determined [28].

### Data Analysis

The abundance of *Enterococcus* spp. cells was calculated on the basis of qPCR results as follows: assuming that 1 base pair (bp) of double-stranded DNA is equal to 660 Da (1 Da = 1.66 ×10^−15 ^ng in the metric system), 1 bp is equal to 1.095×10^−12 ^ng. Since amplicon size is 91 bp, one copy of the amplicon corresponds to 0.0996×10^−9^ng DNA. Considering that each enterococcal cell contains 4 copies of 23S rDNA [29], each cell contains 0.3984×10^−9 ^ng of the 23S rDNA target sequence. The enterococcal abundance in the amplified samples was then calculated by the following formula: amplicon weight (ng)/(0.0996×10^−9^×4). Although it is well known that multiple copies of enterococcal 23S rDNA are found in the *Enterococcus* genome (*E. faecalis* and *Enterococcus faecium* contain 4 and 6 copies, respectively), the number of 23S rRNA gene copies per genome has not been determined in all species. The use of 4 copy numbers for qPCR analyses of enterococcal populations in marine samples may introduce a bias, potentially affecting assay accuracy; however, it is currently used worldwide for qPCR determinations involving enterococci [29], thus allowing comparisons with other studies.

Final counts were expressed as cells/g of wet sediment.

One-way analysis of variance (ANOVA) was used to test for differences in the abundance of benthic enterococci in sediments before and after antibiotic exposure. Differences were considered significant at *P* values <0.05.

## Results

### Sediment Analysis

Sediment samples were collected from three stations: an area used for feed administration (St. 1), an area in the same pond located 20 m downstream of the feeding area (St. 2), and an area upstream of the farm that was therefore not influenced by aquaculture activities (St. 3). Major differences in the main environmental characteristics were noted in sediments from the three stations, but especially between those from the farm (St. 1 and St. 2) and those from the control station. The former were dominated by the silt–clay fraction (<63 µm, 93% and 89%) and characterized by a very high organic matter content (28.3 mg/g and 24.3 mg/g), whereas the latter were characterized by a lower percentage of silt-clays (73%) and a lower organic matter concentration (13.4 mg/g).

### Optimization of qPCR for the Enumeration of Enterococci

The qPCR assay developed in this study showed very high reproducibility and repeatability. Intra- and interassay reproducibility were both very satisfactory; CVs were 1.8% (at the concentration of 10^−6^ ng of the target gene), 1.4% (10^−7^ ng), 0.5% (10^−8^ ng), and 1.2% (10^−9^ ng) for intra-assay and 1.0% (of 10^−6^ ng), 1.3% (10^−7^ ng), 3.4% (10^−8^ ng), and 1.5% (10^−9^ ng) for interassay comparisons. The LOD of the qPCR assay was 5.225×10^−9 ^ng, corresponding to 52 copies of the 23S rRNA gene of *E. faecalis* ATCC 29212 (reference strain), corresponding to 13 cells. The qPCR showed a linear dynamic range over the DNA concentrations tested for the target gene. The average efficiency of the qPCR reaction was 109.2%, while the average regression coefficient of R^2^ was 0.97. Melt curve analysis showed a clear and reproducible melting peak between 80.5 and 81°C.

### Enumeration of Enterococci

The abundance of culturable enterococci estimated with the MF technique was respectively 1.12×10^2^, 1.00×10^2^, and 0.12×10^2^ CFU/g in sediments from St. 1, St. 2, and St. 3, whereas total enterococcal abundance estimated by qPCR was respectively 5.03×10^5^, 1.69×10^5^, and 5.70×10^5^ cells/g.

**Figure 2:**
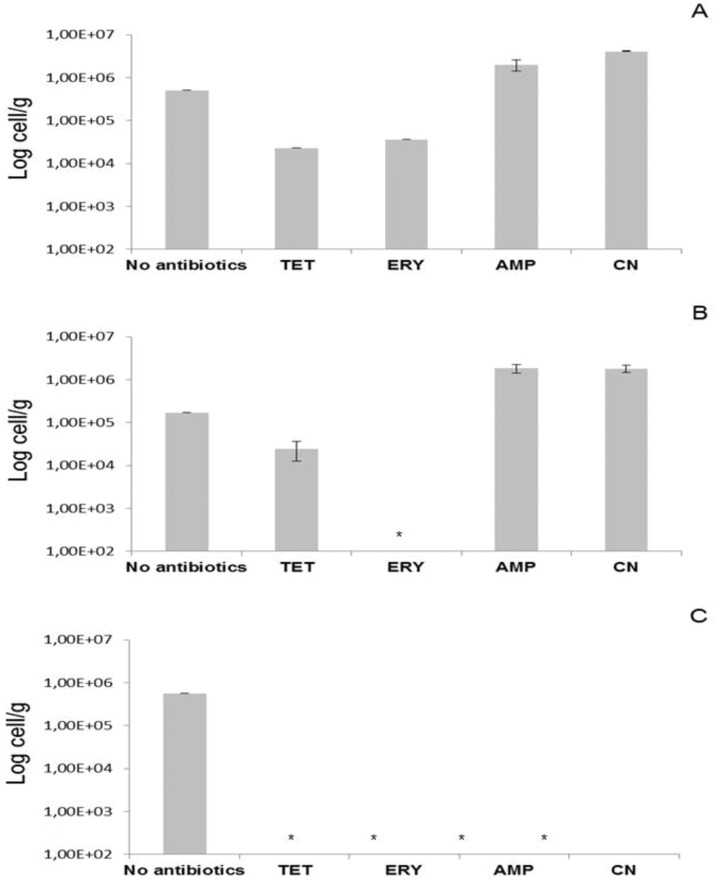
Abundance of benthic enterococci. Enterococcal abundance in the farm sediments and at the control site was determined by qPCR before and after incubation with antibiotic-supplemented BHI broth. A, St. 1; B, St. 2 and C, St. 3. *Not detectable i.e.<LOD of the qPCR assay.

The same sediments were analyzed by qPCR after *in vitro* incubation with antibiotic to assess the response of enterococci to selective pressure and the effect of this exposure on the AR gene profile of the sediment. The abundance of benthic enterococci in sediments collected inside the farm increased significantly in presence of AMP and CN, but not of TET and ERY (Figure 2). The increase was 4 (AMP) and 8 times (CN) (p<0.01) in samples from St. 1, and 11 times (AMP and CN; p<0.01) in those from St. 2. Enterococcal abundance did not increase in sediments from St. 3 (Figure 2).

### Antibiotic Susceptibility Testing of the Isolated Enterococci

A total of 476 isolates (225 from St. 1, 228 from St. 2, and 23 from St. 3) were recovered with the MF technique, and 250 were streaked on SB medium; 150 cultures showing good growth were used in antibiotic susceptibility tests. Neither MICs above the resistance breakpoint nor high-level resistance to CN was detected.

### Direct Detection of Resistance Genes, before and after Antibiotic Enrichment

We sought the TET, macrolide, AMP, and aminoglycosides resistance genes more frequently detected in enterococci. Two newly-developed multiplex PCRs were used to detect directly selected TET [*tet*(M), *tet*(L) and *tet*(O)] and macrolide [*erm*(B), *erm*(A) and *mef*] resistance genes in sediment samples; AMP (*blaZ*) and aminoglycoside [(*aac (6′)-Ie aph (2*″*)-Ia*] resistance genes were sought using individual assays. *tet*(M) and *tet*(L) were detected at all 3 stations, whereas genes coding for ERY, AMP and CN resistance were never found (Table 3). No difference in the AR gene profiles was seen after incubation with AMP and CN, while considerable changes were observed after incubation with TET and ERY. After growth in presence of TET, *tet*(L) and *tet*(M) were no longer detectable in any sample nor in those from St. 3, respectively; *tet*(O) became detectable in the sample from St. 2. Incubation with ERY resulted in detection of *erm*(B) in sediments from St. 1 and St. 2 and of *mef* in those from St. 1 and St. 3.

**Table 3:** Resistance genes detected before and after sediment incubation in antibiotic-supplemented BHI broth.

Station	Resistance genes							
	Before antibiotic exposure				After antibiotic exposure*			
	TET	ERY	AMP	CN	TET	ERY	AMP	CN
St.1	*tet*(M),*tet*(L)	–	–	–	*tet*(M)	*erm*(B),*mef*	–	–
St.2	*tet*(M),*tet*(L)	–	–	–	*tet*(M),*tet*(O)	*erm*(B)	–	–
St.3	*tet*(M),*tet*(L)	–	–	–	–	*mef*	–	–

* detected both in sediment and in broth.

## Discussion

Enterococci are among the major etiological agents of hospital-associated infections [30]. They are characterized by a proneness to acquire resistance determinants [31] and by rapid adaptation to environmental conditions [11], [32], [33]. Aquaculture is believed to contribute to the spread and persistence of AR in the environment and indeed antibiotic-resistant bacteria have frequently been detected at aquaculture sites [4], [34]. The study of resistant enterococci in sediment under aquaculture farms has the potential to disclose important information about the ecology of these bacteria in habitats outside the normal host and about the environmental factors, that can contribute to their evolution in the marine environment.

Several studies have addressed the dynamics and abundance of enterococci in seawater and sediment using different approaches, including MF and molecular assays based on qPCR [29], [35] and RT-qPCR [8], [36]. However, little is known of the impact of fish farms on the origin and spread of antibiotic-resistant strains and related AR genes [4], [23]. We found higher counts of benthic enterococci with qPCR than with culture methods in line with previous data showing that cultivation-based techniques underestimate bacterial abundance in marine samples, due to large amounts of nonculturable cells [8]. Furthermore, the finding of a greater amount of culturable enterococci within the farm than in control sediments indicates that benthic enterococci under aquaculture sites may be more metabolically active. This could depend on large inputs of labile organic nutrients connected with farming activities, described by other researchers [37] and found in the present work, where the concentration of sedimentary organic matter and the silt–clay fraction found in the breeding pond were greater than the one determined in the control station. Since enterococci are part of the fish gut microbiota [38], the accumulation of fecal matter in the sediment beneath the fish farm could also directly contribute to the amount and diversity of the enterococci recovered from the farm.

Different environmental factors may have influenced the discrepancies in enterococcal counts found between samples collected inside and outside the farm. Local bird populations feeding on aquaculture might be involved in delivery of fecal material, with its burden of intestinal, possibly antibiotic-resistant, enterococci [39]. Local waste impacting the various sites differently may also be implicated, although no landfills are found close to the farm area. A contribution from bird fecal material cannot of course be excluded, but it is probably an inherent risk factor in fish farms.

This study focused on assessing the presence of antibiotic-resistant enterococci, to gain insights into the impact of fish-farming activities on the presence and spread of resistant strains.

The lack of use of antibiotics, declared by the owner of the farm, does not contrast with our results. Indeed no antibiotic-resistant enterococcal strains were isolated before the antibiotic-enrichment step, while in a farm where antibiotics had not been used over only the previous two years we recovered 12% of resistant strains [4].

Antibiotic exposure induced considerable changes in the abundance of benthic enterococci in farm (St. 1 and St. 2) compared to control sediments (St. 3). AMP and CN clearly favored enterococcal growth in farm sediments, as demonstrated by the fact that the number of bacteria detected after antibiotic exposure exceeded the one obtained before exposure; in contrast, St. 3 samples were qPCR-negative, yielding enterococcal counts below the method’s sensitivity threshold. Exposure to TET stimulated enterococcal growth in samples from both farm stations; ERY exerted a similar effect on St. 1 sediments, despite the fact that the number of enterococci was lower there than in untreated samples. Even though direct counts cannot of course be compared with counts performed after growth in rich medium, the comparison may nonetheless provide indirect evidence of the abundance of resistant bacteria in the original sediments. The low counts obtained after exposure to TET and ERY can be explained by the presence of a small fraction of enterococci resistant to these antibiotics or by the slow growth rate of resistant bacteria.

None of the enterococcal isolates was resistant to any of the antibiotics tested, including TET; however, *tet*(M) and *tet*(L) were detected by direct PCR in sediments from all sites before antibiotic enrichment. This is not surprising, because *tet* genes are widely disseminated in the environment even in the absence of antimicrobial use [4], [40]; moreover, an environmental evolution of TET resistance has recently been suggested [41]. The lack of TET-resistant enterococci in any of our sediment samples may probably be explained by considering that *tet* genes are also carried by non-enterococcal strains, including autochthonous marine bacteria. The presence of dormant bacteria, including enterococci, is a further possibility that could moreover explain the *ex novo* detection of *tet*(O), *erm*(B), and *mef* after incubation in antibiotic-supplemented rich medium, a source of readily available nutrients which coupled to incubation at 37°C may have provided a suitable environment for bacterial reactivation and growth.

Differences in resistance gene profiles before and after exposure to antibiotics and rich medium were particularly evident in the sediments collected under the farm (St. 1 and St. 2) incubated with ERY. Erythromycin may have selected and revived macrolide-resistant bacteria, making macrolide resistance genes, i.e. *erm*(B) and *mef*, detectable by PCR. AR gene profiles were also altered after incubation with TET, with *ex novo* detection of *tet*(O) and loss of *tet*(L), whereas *tet*(M) was uniformly detected. These findings may be explained by the fact that whereas *tet*(O) and *tet*(M) are ribosomal protection genes conferring high-level resistance, *tet*(L) codes for antibiotic efflux, which is characterized by a low-level of resistance. The present data indicate a possible contribution of aquaculture practices to the selection of genetic determinants conferring high-level resistance. The failed detection of *erm*(B) in sediments from the control station seems to corroborate this hypothesis.

Despite being limited to a single farm, our data indicate that aquaculture environments may not only select for resistant strains when using antibiotics, as reported previously [2], [42], but also influence the metabolic activity of benthic enterococci due to the abundance of organic carbon sources. Since the owner denied all antibiotic use and we found no resistant strains before the enrichment step, these data suggest that aquaculture may constitute a reservoir of resistance genes irrespective of antibiotic use. This view is supported by the data obtained from the control station, where the lack of enterococci after antibiotic exposure is consistent with the lower abundance of resistant strains outside than inside the farm.

In conclusion, our findings indicate that aquaculture has the potential to affect antibiotic-resistant benthic enterococcal populations and that fish-farm sediments can contain AR genes of putative enterococcal origin. Moreover, besides hosting active and culturable strains, fish-farm sediments could constitute reservoirs of dormant resistant enterococci capable of quick reactivation following new nutrient inputs into the system. The hypothesis agrees with recent studies suggesting that dormancy generates a seed bank, i.e. a reservoir of dormant bacteria that can eventually revive under different environmental conditions [43]. The antibiotic-resistant enterococcal seed bank found in fish-farm sediments might constitute an underrated health risk and stresses the need for long-term monitoring of the effects of aquaculture operations on potentially pathogenic microbes, to assess their antimicrobial resistance properties and the potential for their spread and transmission to different bacterial species.
